# The prognostic value of a combined immune score in tumor and immune cells assessed by immunohistochemistry in triple-negative breast cancer

**DOI:** 10.1186/s13058-023-01710-8

**Published:** 2023-11-03

**Authors:** Ji Eun Choi, Jae Seok Lee, Min-Sun Jin, Ilias P. Nikas, Kwangsoo Kim, Sunah Yang, Soo Young Park, Jiwon Koh, Sohyeon Yang, Seock-Ah Im, Han Suk Ryu

**Affiliations:** 1 Department of Pathology, Chungnam National University Sejong Hospital, Sejong, Republic of Korea; 2 Department of Pathology, Samsung Changwon Hospital, Sungkyunkwan University School of Medicine, Changwon, Republic of Korea; 3 Department of Pathology, Bucheon St. Mary’s Hospital, College of Medicine, The Catholic University of Korea, Bucheon, Republic of Korea; 4 School of Medicine, European University Cyprus, Nicosia, Cyprus; 5 Transdisciplinary Department of Medicine and Advanced Technology, Seoul National University Hospital, Seoul, Republic of Korea; 6 Department of Pathology, Seoul National University College of Medicine, 103 Daehak-ro, Jongno-gu, Seoul, 03080 Republic of Korea; 7 Department of Internal Medicine, Seoul National University Hospital, Seoul National University College of Medicine, 103 Daehak-ro, Jongno-gu, Seoul, 03080 Republic of Korea; 8 Cancer Research Institute, Seoul National University, Seoul, Republic of Korea; 9 Translational Medicine, Seoul National University College of Medicine, 103 Daehak-ro, Jongno-gu, Seoul, 03080 Republic of Korea; 10 Pharmonoid Co., Ltd., Seoul, Republic of Korea

**Keywords:** Triple-negative breast cancer (TNBC), Immune checkpoint proteins, Prognostic model, Combined immune score, Programmed death ligand 1 (PD-L1), Overall survival (OS), Progression-free survival (PFS)

## Abstract

**Background:**

This study aimed to develop a novel combined immune score (CIS)-based model assessing prognosis in triple-negative breast cancer (TNBC).

**Methods:**

The expression of eight immune markers (PD-1, PD-L1, PD-L2, IDO, TIM3, OX40, OX40L, and H7-H2) was assessed with immunohistochemistry on the tumor cells (TCs) and immune cells (ICs) of 227 TNBC cases, respectively, and subsequently associated with selected clinicopathological parameters and survival. Data retrieved from The Cancer Genome Atlas (TCGA) were further examined to validate our findings.

**Results:**

All immune markers were often expressed in TCs and ICs, except for PD-1 which was not expressed in TCs. In ICs, the expression of all immune markers was positively correlated between one another, except between PD-L1 and OX40, also TIM3 and OX40. In ICs, PD-1, PD-L1, and OX40L positive expression was associated with a longer progression-free survival (PFS; *p* = 0.040, *p* = 0.020, and *p* = 0.020, respectively). In TCs, OX40 positive expression was associated with a shorter PFS (*p* = 0.025). Subsequently, the TNBC patients were classified into high and low combined immune score groups (CIS-H and CIS-L), based on the expression levels of a selection of biomarkers in TCs (TCIS-H or TCIS-L) and ICs (ICIS-H or ICIS-L). The TCIS-H group was significantly associated with a longer PFS (*p* < 0.001). Furthermore, the ICIS-H group was additionally associated with a longer PFS (*p* < 0.001) and overall survival (OS; *p* = 0.001), at significant levels. In the multivariate analysis, both TCIS-H and ICIS-H groups were identified as independent predictors of favorable PFS (*p* = 0.012 and *p* = 0.001, respectively). ICIS-H was also shown to be an independent predictor of favorable OS (*p* = 0.003). The analysis of the mRNA expression data from TCGA also validated our findings regarding TNBC.

**Conclusion:**

Our novel TCIS and ICIS exhibited a significant prognostic value in TNBC. Additional research would be needed to strengthen our findings and identify the most efficient prognostic and predictive biomarkers for TNBC patients.

**Supplementary Information:**

The online version contains supplementary material available at 10.1186/s13058-023-01710-8.

## Introduction

Immune checkpoint inhibitors (ICIs), which block checkpoint molecules such as the cytotoxic T lymphocyte antigen-4 (CTLA-4), programmed death 1 (PD-1) and programmed death ligand 1 (PD-L1), have become a standard therapeutic option across several types of malignant tumors [1, 2].

Many recent studies have shown that triple-negative breast cancer (TNBC) is highly immunogenic, exhibiting a better response to ICIs than other molecular subtypes of breast cancer [3, 4]. Lately, a combination therapy of a monoclonal antibody targeting PD-1 (e.g., pembraolizumab) plus chemotherapy has been approved by the US Food and Drug Administration (FDA) for patients with locally recurrent unresectable or metastatic TNBC, given that the tumors express PD-L1 [5]. Nevertheless, the therapeutic efficacy of ICI monotherapy still remains suboptimal, ranging from 5 to 23% in TNBC [4].

T cell-mediated immunity includes multiple sequential steps. The ultimate amplitude and quality of the response, which is initiated through the antigen recognition of ligand–receptor interactions, is regulated by a balance between co-stimulatory and inhibitory signals. Examples of co-stimulatory molecules include the OX40 and OX40L, which are expressed by T cells and antigen-presenting cells. Co-inhibitory signaling occurs through interactions between receptors, such as the TIM-3 expressed on T cells, and their ligands expressed on antigen-presenting cells and other cells of the microenvironment [6]. Along with the receptor-ligand interactions, immune checkpoint enzymes are also important response regulators. For instance, the IDO (Indoleamine 2,3-dioxygenase) inhibits immune cell effector functions and/or facilitates T cell death, showing immunotherapeutic efficacy in solid cancers [7]. Therefore, multiple additional immune checkpoints represent promising targets for therapeutic blockade [8]. Unlike initial trials with ICIs as monotherapy, recent trials evaluating a combination of agonists of co-stimulatory receptors and/or antagonists of inhibitory signals outnumber single-agent trials [9].

Recent state-of-the-art technologies, including single-cell RNA sequencing and multiplex immunohistochemistry, have revealed the complexity of heterogenous tumor-infiltrating immune cells influencing the response to immunotherapy [10]. Therefore, more knowledge on the interaction of immunoregulatory proteins between the tumor microenvironment immune and breast cancer cells is required to overcome the tumor’s ability to evade immunotherapy. Accumulating research aimed to discover next-generation immune checkpoint targets has been ongoing [11, 12], while numerous clinical studies have shown promise regarding such biomarkers’ application to future oncology practice [13].

Along with the therapeutic aspects of ICIs, the expression of immune checkpoints has additionally been associated with the prognosis of various cancer types [14–16]. The prognostic models using immune gene panel or mRNA scoring systems have also been suggested in several malignant tumor types, based on patient data derived from The Cancer Genome Atlas (TCGA) repository [17, 18]. Indeed, high-throughput genetic tests are widely implemented in clinical practice; however, immunohistochemistry is still considered as a standard diagnostic tool for tumor classification and therapeutic decision [19]. In breast cancer, immunohistochemical testing has also been crucial for precision medicine, since the St. Gallen consensus guidelines recommend it as a standard diagnostic, prognostic, and predictive tool [20, 21]. The recent implementation of companion diagnostics for PD-L1 or PD-1 detection is also based on immunohistochemistry. However, in contrast to other cancer types, the prognostic role of ICIs-related immunohistochemistry has still been considered ambiguous in TNBC.

The purpose of this study was to identify novel checkpoints and define aggressive immune-phenotype subgroups in TNBC. We selected eight immune checkpoint targets previously shown to exhibit clinical significance in various cancers [22–27] and evaluated their protein expression with immunohistochemistry in the tumor cells (TCs) and immune cells (ICs) of a well-characterized TNBC cohort [28, 29]. We further developed a novel “combined immune score (CIS)”-based classification, which resulted in distinct groups predicting the clinical outcome of TNBCs.

## Methods

### Patient selection and study design

A total number of 306 TNBC patients who received surgical resection after being diagnosed with invasive ductal carcinoma at Seoul National University Hospital between 2003 and 2006 were included in this study. Histologic grading was based on the Nottingham grading system [30]. Selected clinicopathologic parameters and patient survival data were retrieved from the electronic medical records system. These included the following: age, tumor size, nuclear grade, histologic grade, presence/absence of a ductal carcinoma in situ component, lymphovascular invasion or lymph node metastasis, in addition to each patient’s anatomical (pTNM) and prognostic stage, according to the 8th edition of the American Joint Committee on Cancer (AJCC) [31]. Patients who had received neoadjuvant chemo- or radiotherapy were excluded from further analysis.

All enrolled TNBC cases had previously been subjected to estrogen receptor (ER) and progesterone receptor (PR) immunohistochemical analysis, also to Human epidermal growth factor receptor 2 (HER2) immunohistochemical and/or in situ hybridization testing, and had been evaluated based on the 13th St. Galen International Breast Cancer Conference and ASCO/CAP guidelines [20, 32, 33]. ER (1:100, 1D5; Novocastra Laboratories, Newcastle, UK) and PR (1:200, PgR636; Dako, Glostrup, Denmark) expression was considered as positive when ≥ 1% of staining was observed in tumor cells, according to the 2010 ASCO/CAP guidelines [32]. HER2 immunohistochemistry (1:1, 4B5; Ventana, Medical System, Tucson, AZ, USA) was regarded as positive (3+) when ≥ 10% of tumor cells showed complete and intense circumferential membranous staining, based on 2018 ASCO/CAP guidelines [33]. In equivocal cases (2+), fluorescence in situ hybridization (FISH) using the PathVysion assay (Abbott Molecular, Downers Grove, IL) was performed, and the test was considered as positive when either a ≥ 2.0 ratio of the HER2 to chromosome 17 gene copy number or ≥ 6.0 of average HER2 signals per tumor cell were observed [33]. This study was approved by the Institutional Review Board of Seoul National University Hospital (IRB No. 1511-085-720).

### Immunohistochemistry and interpretation

Immunohistochemical staining was performed on 227 TNBC cases, arranged in tissue microarrays consisting of 2 mm cores from the aforementioned cases (Superbiochips Laboratories, Seoul, Korea). A 4-μm section from each block was subjected to immunohistochemistry, using the Benchmark automatic immunostaining device (Ventana, Arizona, USA). The following eight primary antibodies were used: PD-1 (PDCD1) (1:20; Cell Marque, California, USA), PD-L1 (B7-H1) (1:100; Cell Signaling, Massachusetts, USA), PD-L2 (B7-DC) (1:500; Sigma-Aldrich, Missouri, USA), IDO (IDO1) (1:30, 1L30; Millipore-Sigma, Massachusetts, USA), TIM3 (HAVCR2) (1:550; Abbexa Ltd, Cambridge, UK), OX40 (TNFRSF4) (1:125; Novus Biologicals, Colorado, USA), OX40L (TNFSF4) (1:30; Millipore-Sigma, Massachusetts, USA), and B7-H2 (ICOSLG) (1:300; Novus Biologicals, Colorado, USA). Interpretation of all eight biomarkers was based on both intensity and proportion of the positively stained TCs and ICs; the latter included lymphocytes, macrophages, dendritic cells, and granulocytes [34]. For each case, the intensity score (IS) of each immune marker was graded as follows: 0 (negative), 1 (weak), 2 (moderate), and 3 (strong). Furthermore, the proportion score (PS) was graded as follows: 0 (stain under 1%), 1 (1–5%), 2 (5–10%), 3 (10–25%), 4 (25–50%), and 5 (> 50%). Subsequently, the IS and PS of each biomarker were multiplied to generate a final score, ranging from 0 to 15. To determine its clinicopathologic and prognostic significance, each marker’s expression score of 1 or greater (IS × PS ≥ 1) was regarded as positive when evaluating either TCs or ICs, similar to previous studies that considered as positive the expression of at least 1% of cells with any intensity [35, 36]. All immunohistochemical stainings were interpreted independently by three experienced breast pathologists (H.S.R., M.S.J., and J.E.C.) to enhance accuracy, while any discordance among them was resolved with a consensus.

### Validation of combined immune score using the TCGA database

To validate our findings, the TCGA breast cancer dataset was used to investigate the mRNA expression of the ICI-related markers exhibiting prognostic relevance in our immunohistochemical analysis. The expression of each biomarker was considered as positive or negative, using the 25th percentile as a cut-off. We also classified all breast cancer and TNBC cases into two groups, based on the gene expression levels of a combination of immune markers, similar to our immunohistochemical analysis. Kaplan–Meier curves and log-rank tests were used to determine whether there was a significant difference in survival. The TCGA data was obtained from cBioPortal and the survival analysis was processed using the R software, version 4.1.0 (R Foundation for Statistical Computing, Vienna, Austria).

### Statistical analysis

The chi-square and Pearson’s correlation coefficient tests were utilized to investigate the potential association of the expression of the abovementioned eight immune markers in TCs and ICs with the extracted clinicopathological variables and between each other, respectively. To investigate the effect of each immune marker expression on TNBC patients’ progression-free survival (PFS)—measured from the date of surgery to the date of local recurrence and/or distant metastasis—and overall survival (OS), the Kaplan–Meier analysis with log-rank test was performed. The Cox proportional hazard model was further adapted to conduct multivariate analysis and identify independent prognostic predictors. Clinicopathologic characteristics were adjusted using a backward stepwise model, including covariates with a prognostic role. Statistical significance was considered at the level of *p* < 0.05. To construct each CIS, we only selected immune markers exhibiting a *P*-value of less than 0.1 (*p* < 0.1) in the PFS Kaplan–Meier analysis [37]. Statistical analysis was performed with the SPSS software for Windows, version 22.0 (SPSS, Chicago, IL, USA).

## Results

### Patient characteristics and immune marker expression

The clinicopathologic characteristics of the enrolled patients are shown in Table 1. All patients were female with a median age of 48 (range: 21–76) years. Most of the patients were under 60 years old (86.3%) at the time of diagnosis. There were 145 (63.9%) patients with a tumor diameter greater than 2.0 cm, while 73 (32.2%) patients showed lymph node metastasis. Most TNBCs had nuclear score 3 (80.2%), also histologic grade 3 (82.4%), according to the Nottingham histologic grade. The anatomic stage was I in 61 (26.9%), II in 128 (56.4%), and III in 38 (16.7%) patients. The prognostic stage was II in 62 (27.3%) and III in 165 (62.7%) patients. The median follow-up duration was 103 months, and 217 (95.6%) of the tested TNBC patients had received adjuvant chemotherapy.

**Table 1:** Clinicopathologic characteristics of triple-negative breast cancer (TNBC)

Characteristics	n (%)
*Age (years)*	
< 60	196 (86.3%)
≥ 60	31 (13.7%)
*Tumor size (cm)*	
≤ 2	82 (36.1%)
> 2	145 (63.9%)
*Nuclear score*	
Score 1	0 (0%)
Score 2	45 (19.8%)
Score 3	182 (80.2%)
*Histologic grade*	
Grade 1	0 (0%)
Grade 2	40 (17.6%)
Grade 3	187 (82.4%)
*Ductal carcinoma in situ*	
Absent	96 (57.7%)
Present	131 (42.3%)
*Lymphovascular invasion*	
Absent	92 (40.5%)
Present	135 (59.5%)
*Lymph node metastasis*	
Absent	154 (67.8%)
Present	73 (32.2%)
*Anatomic stage*	
Stage I	61 (26.9%)
Stage II	128 (56.4%)
Stage III	38 (16.7%)
*Prognostic stage*	
Stage I	0 (0%)
Stage II	62 (27.3%)
Stage III	165 (72.7%)

**Fig. 1:**
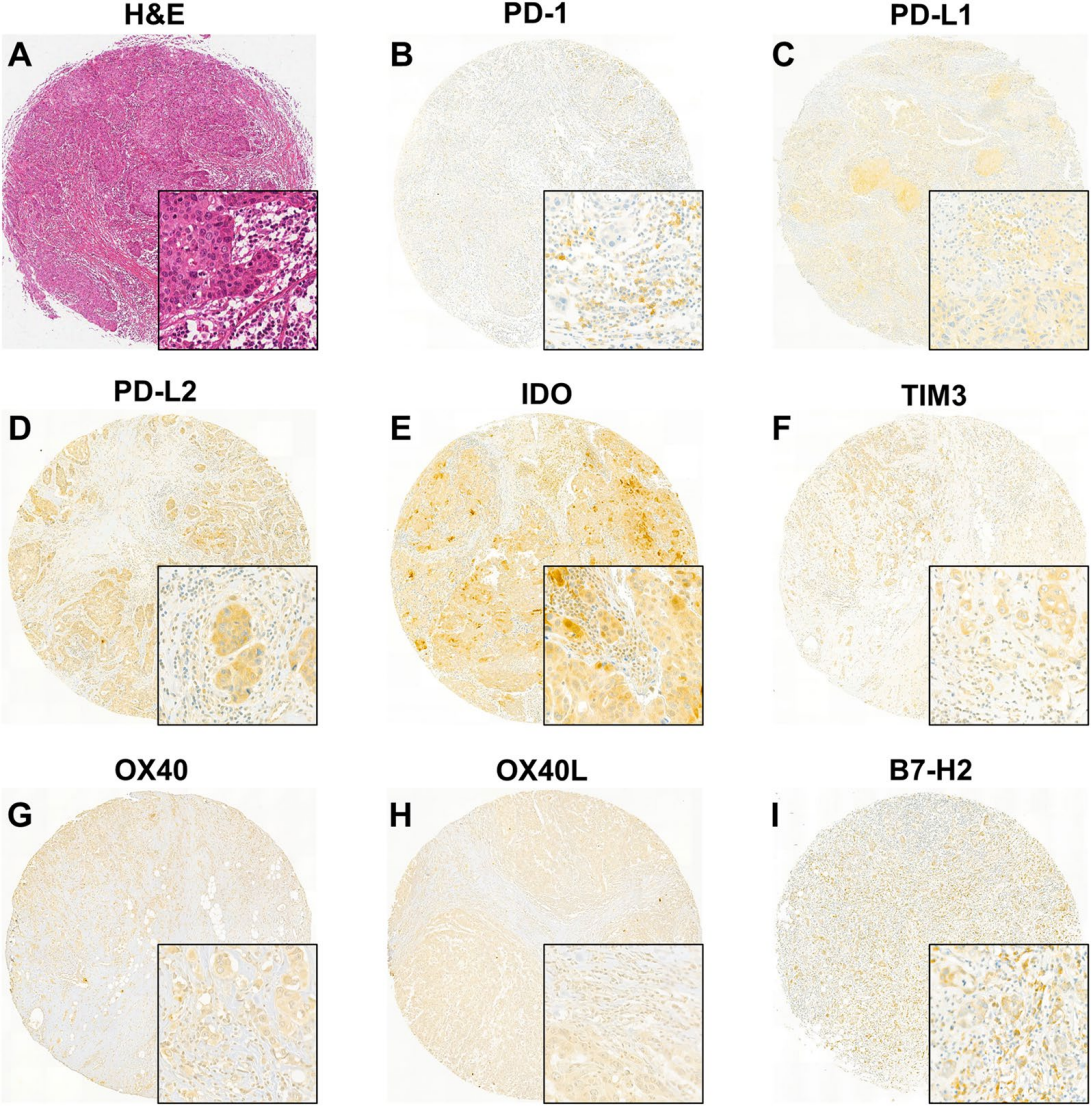
Representative H&E and immunohistochemistry images. Among the immune markers tested, PD-1 is expressed in immune cells only. Expression of the other immune markers is shown both in tumor cells and immune cells. **A** H&E, **B** PD-1, **C** PD-L1, **D** PD-L2, **E** IDO, **F** TIM3, **G** OX40, **H** OX40L, and **I** B7-H2. (Scan view; inlets: × 400 magnification)

**Fig. 2:**
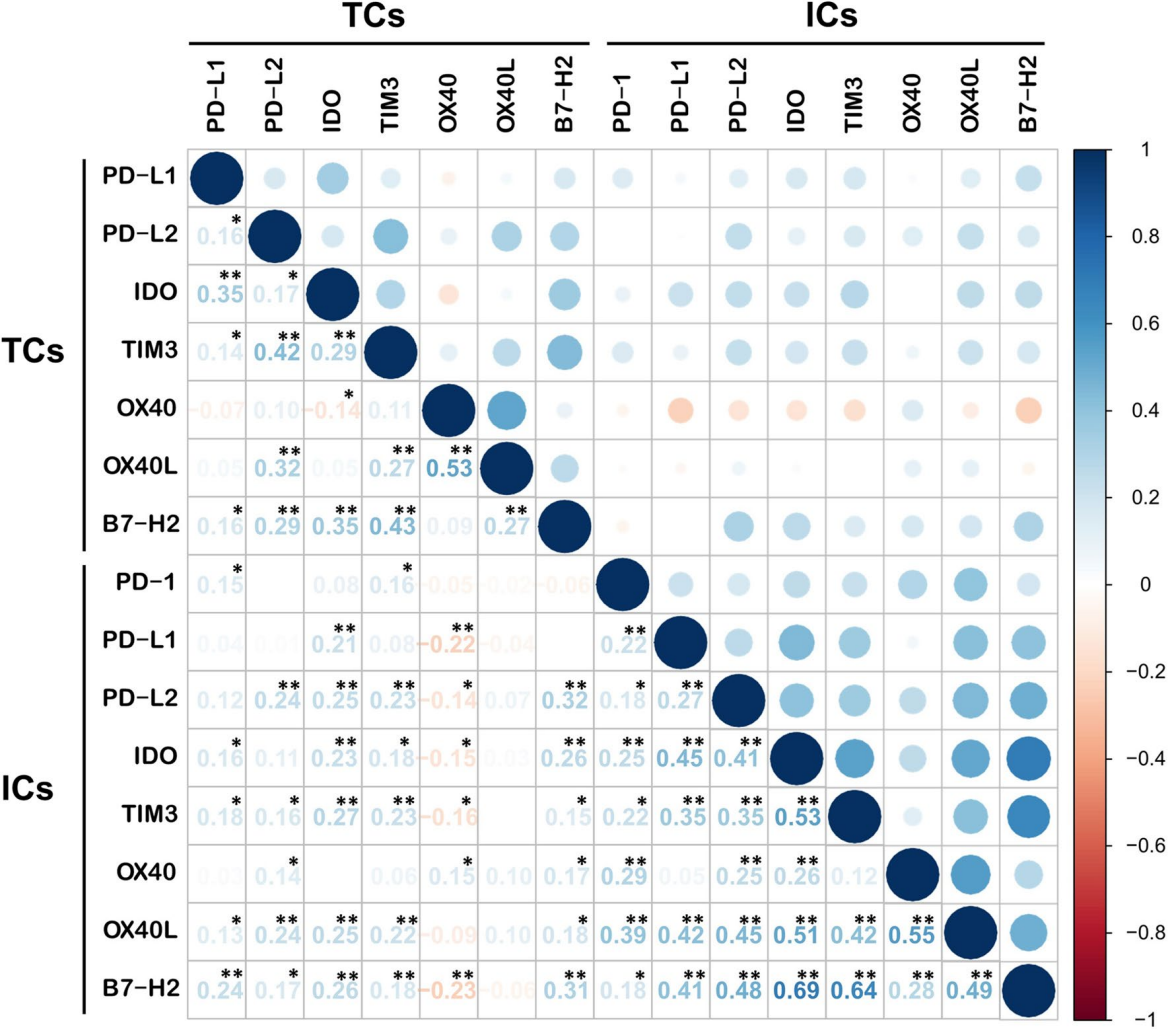
Correlation matrix regarding the expression of all 8 immune markers tested in tumor cells (TCs) and immune cells (ICs). Pearson correlation coefficients are presented in text (blue, positive correlations or red, negative correlations). Significance levels are indicated by an asterisk: *p < 0.05, **p < 0.01

Expression of each immune marker was evaluated separately in TCs and ICs (Fig. 1). In TCs, positive expression was identified in the following markers: PD-L1 (n = 46, 20.3%), PD-L2 (n = 197, 86.8%), IDO (n = 129, 56.8%), TIM3 (n = 109, 48.0%), OX40 (n = 82, 36.1%), OX40L (n = 86, 37.9%), and B7-H2 (n = 193, 85.0%). PD-1 was not expressed in TCs in all TNBC cases examined. Positive expression in ICs was observed in the following markers: PD-1 (n = 126, 55.5%), PD-L1 (n = 91, 40.1%), PD-L2 (n = 108, 47.6%), IDO (n = 154, 67.8%), TIM3 (n = 104, 45.8%), OX40 (n = 84, 37.0%), OX40L (n = 103, 45.4%), and B7-H2 (n = 150, 66.1%). The expression of the eight immune markers were compared between one another, as shown in Fig. 2. In ICs, the expression of each biomarker was positively correlated with the rest at a significant level, except between PD-L1 and OX40, also TIM3 and OX40. On the other hand, in TCs, a significant positive correlation was also shown—especially between PD-L2 and TIM3 (*p* < 0.01, rho = 0.42), TIM3 and B7-H2 (*p* < 0.01, rho = 0.43), and OX40 and OX40L (*p* < 0.01, rho = 0.53)—albeit less often than in ICs. Notably, IDO expression in TCs was negatively correlated with the OX40 expression, at a significant level (*p* = 0.04, rho =− 0.14).

### Comparison of each immune marker expression with clinicopathologic parameters

Furthermore, the immunohistochemical expression of the eight immune markers in both TCs and ICs was compared with the extracted clinicopathologic parameters (Table 2). Regarding TCs, PD-L1 positive expression was significantly associated with an anatomic stage I and II, compared to stage III (*p* = 0.038). OX40 positive expression was associated with a higher rate of lymph node metastasis (*p* = 0.011) and an advanced anatomic stage (*p* < 0.001), at significant levels. OX40L positivity was also associated with an advanced anatomic stage (*p* = 0.016). Regarding ICs, PD-L1 positive expression was associated with an anatomic stage I and II, compared to stage III (*p* = 0.024), which was a concordant finding with the result of TCs. TIM3 and OX40L positive expression was significantly associated with lymph node metastasis (*p* = 0.031 and *p* = 0.040). OX40L positivity was also significantly associated with an advanced anatomic stage (*p* = 0.003). In our analysis, OX40 was the only immune marker that was significantly associated with an aggressive tumor behavior, such as a higher risk of lymph node metastasis and an advanced anatomic stage both in TCs and ICs (in TCs: *p* = 0.011 and *p* < 0.001; in ICs: *p* = 0.040 and *p* = 0.003, respectively). In addition, when positive expression in both TCs and ICs was considered to assign the expression of an immune marker as positive, OX40 positive expression was significantly associated with the presence of lymph node metastasis and advanced anatomic stage (*p* = 0.005 and *p* < 0.001, respectively), in contrast to other immune markers (Additional file 1: Table S1).

**Table 2:** Expression of the tested immune-related markers separately in tumor cells and immune cells, and its clinicopathological significance

Expression of immune markers	Lymph node metastasis				Anatomic stage				Prognostic stage			
	Absent (N = 154)	Present (N = 73)		*P* value	I&II (N = 189)	III (N = 38)		*P* value	I&II (N = 62)	III (N = 165)		*P* value
*In tumor cells*												
*PD-1*				–				–				–
Positive (N = 0)	0 (0.0%)	0 (0.0%)			0 (0.0%)	0 (0.0%)			0 (0.0%)	0 (0.0%)		
Negative (N = 227)	154 (67.8%)	73 (32.2%)			189 (83.3%)	38 (16.7%)			62 (27.3%)	165 (72.7%)		
*PD-L1*				0.779				**0.038**				0.342
Positive (N = 46)	32 (69.6%)	14 (30.4%)			43 (93.5%)	3 (6.5%)			10 (21.7%)	36 (78.3%)		
Negative (N = 181)	122 (67.4%)	59 (32.6%)			146 (80.7%)	65 (19.3%)			52 (28.7%)	129 (71.3%)		
*PD-L2*				0.324				0.299				0.723
Positive (N = 197)	136 (69.0%)	61 (31.0%)			166 (84.3%)	31 (15.7%)			53 (26.9%)	144 (73.1%)		
Negative (N = 30)	18 (60%)	12 (40%)			23 (76.7%)	7 (23.3%)			9 (30.0%	21 (70.0%)		
*IDO*				0.313				0.884				0.711
Positive (N = 129)	84 (65.1%)	45 (34.9%)			107 (82.9%)	22 (17.1%)			34 (26.4%)	95 (73.6%)		
Negative (N = 98)	70 (71.4%)	28 (28.6%)			82 (83.7%)	16 (16.3%)			28 (28.6%)	70 (71.4%)		
*TIM3*				0.091				0.182				0.597
Positive (N = 109)	68 (62.4%)	41 (37.6%)			87 (79.8%)	22 (20.2%)			28 (25.7%)	81 (74.3%)		
Negative (N = 118)	86 (79.2%)	32 (27.1%)			102 (86.4%)	16 (13.6%)			34 (28.8%)	84 (71.2%)		
*OX40*				**0.011**				** < 0.001**				0.665
Positive (N = 82)	47 (57.3%)	32 (42.7%)			56 (68.3%)	26 (31.7%)			21 (25.6%)	61 (74.4%)		
Negative (N = 145)	107 (73.8%)	38 (23.2%)			133 (91.7%)	12 (8.3%)			41 (28.3%)	104 (71.7%)		
*OX40L*				0.203				**0.016**				0.648
Positive (N = 86)	54 (29.1%)	32 (37.2%)			65 (75.6%)	21 (24.4%)			22 (25.6%)	64 (74.4%)		
Negative (N = 141)	100 (70.9%)	41 (62.8%)			124 (87.9%)	17 (12.1%)			40 (28.4%)	101 (71.6%)		
*B7-H2*				0.671				0.878				0.257
Positive (N = 193)	132 (68.4%)	61 (31.6%)			161 (83.4%)	32 (16.6%)			50 (25.9%)	143 74.1%)		
Negative (N = 34)	22 (64.7%)	12 (35.3%)			28 (82.4%)	6 (17.6%)			12 (35.3%)	22 (64.7%)		
*In immune cells*												
*PD-1*				0.320				0.495				0.282
Positive (N = 126)	82 (65.1%)	44 (34.9%)			103 (81.7%)	23 (18.3%)			38 (30.2%)	88 (69.8%)		
Negative (N = 101)	72 (71.3%)	29 (28.7%)			86 (85.1%)	15 (14.9%)			24 (23.8%)	77 (76.2%)		
*PD-L1*				0.216				**0.024**				0.795
Positive (N = 91)	66 (72.5%)	25 (27.5%)			82 (90.1%)	9 (9.9%)			24 (26.4%)	67 (73.6%)		
Negative (N = 136)	88 (64.7%)	48 (35.3%)			107 (78.7%)	29 (21.3%)			38 (27.9%)	98 (72.1%)		
*PD-L2*				0.519				0.459				0.455
Positive (N = 108)	71 (65.7%)	37 (34.3%)			92 (85.2%)	16 (14.8%)			32 (29.6%)	76 (70.4%)		
Negative (N = 119)	83 (69.7%)	36 (30.3%)			97 (81.5%)	22 (18.5%)			30 (25.2%)	89 (74.8%)		
*IDO*				0.451				0.642				0.536
Positive (N = 154)	102 (66.2%)	52 (33.8%)			127 (82.5%)	29 (17.5%)			44 (28.6%)	110 (71.4%)		
Negative (N = 73)	52 (71.2%)	21 (28.8%)			62 (84.9%)	11 (15.1%)			18 (247%)	55 (75.3%)		
*TIM3*				**0.031**				0.355				0.472
Positive (N = 104)	63 (60.6%)	41 (39.4%)			84 (80.8%)	20 (19.2%)			26 (25.0%)	78 (75.0%)		
Negative (N = 123)	91 (74.0%)	32 (26.0%)			105 (85.4%)	18 (14.6%)			36 (29.3%)	87 (70.7%)		
*OX40*				**0.040**				**0.003**				0.211
Positive (N = 84)	50 (59.5%)	34 (40.5%)			62 (73.8%)	22 (26.2%)			27 (32.1%)	57 (67.9%)		
Negative (N = 143)	104 (72.7%)	39 (27.3%)			127 (88.8%)	16 (11.2%)			35 (24.5%)	108 (75.5%)		
*OX40L*				0.093				0.931				0.079
Positive (N = 103)	64 (62.1%)	39 (37.9%)			86 (83.5%)	17 (16.5%)			34 (33.0%)	69 (67.0%)		
Negative (N = 124)	90 (72.6%)	34 (27.4%)			103 (83.1%)	21 (16.9%)			28 (22.6%)	96 (77.4%)		
*B7-H2*				0.153				0.278				0.992
Positive (N = 150)	97 (64.7%)	53 (35.3%)			122 (81.3%)	29 (18.7%)			41 (27.3%)	109 (72.7%)		
Negative (N = 77)	57 (74.0%)	20 (26.0%)			67 (87.0%)	10 (13.0%)			21 (27.3%)	56 (72.7%)		

*P* values in bold indicate statistical significance (*p* < 0.05)

### Prognostic significance of each immune marker expression in TCs and ICs

During the median follow-up period of 102.6 months, 33 recurrent or metastatic events occurred and 23 patients died with disease. The median PFS and OS were 100.9 and 102.6 months, respectively. The prognostic significance of each individual immune-related marker was analyzed, based on its expression on TCs and ICs (Table 3; Additional file 3: Fig. S1; Additional file 4: Fig. S2). Regarding TCs, OX40 overexpression was associated with a shorter PFS (78.0% vs. 89.7%, *p* = 0.025), while an increased B7-H2 expression had a tendency for shorter PFS (83.4% vs. 97.1%, *p* = 0.054). In contrast, positive expression of PD-L1 in TCs had a tendency of longer PFS (93.5% vs 83.4%, *p* = 0.081). Regarding ICs, positive expression of PD-1 (89.7% vs. 80.2%, *p* = 0.040), PD-L1 (91.3% vs. 80.9%, *p* = 0.020), and OX40L (91.3% vs. 80.6%, *p* = 0.020) were significantly associated with a longer PFS. Additionally, the overexpression of PD-L2 in ICs had a tendency for longer PFS (89.8% vs 81.5%, *p* = 0.086). When positive expression in both TCs and ICs was considered to assign the expression of an immune marker as positive, the positive expression of all eight markers did not show a statistical significance with either PFS or OS (Additional file 2: Table S2).

**Table 3:** Prognostic role of the expression of each immune marker separately in tumor cells and immune cells

Immune marker expressions		PFS (%)	*P* value	OS (%)	*P* value
*In tumor cells*					
PD-1	Negative (N = 227)	85.5	–	92.5	–
	Positive (N = 0)	0		0	
PD-L1	Negative (N = 181)	83.4	0.081	91.7	0.348
	Positive (N = 46)	93.5		95.7	
PD-L2	Negative (N = 30)	90.0	0.494	96.7	0.378
	Positive (N = 197)	84.8		91.9	
IDO	Negative (N = 98)	84.7	0.640	89.8	0.145
	Positive (N = 129)	86.0		94.6	
TIM3	Negative (N = 118)	83.9	0.538	94.1	0.369
	Positive (N = 109)	87.2		90.8	
OX40	Negative (N = 145)	89.7	**0.025**	94.5	0.181
	Positive (N = 82)	78.0		89.0	
OX40L	Negative (N = 141)	87.9	0.224	93.6	0.475
	Positive (N = 86)	81.4		90.7	
B7-H2	Negative (N = 34)	97.1	0.054	94.1	0.782
	Positive (N = 193)	83.4		92.2	
*In immune cells*					
PD-1	Negative (N = 101)	80.2	**0.040**	90.1	0.208
	Positive (N = 126)	89.7		94.4	
PD-L1	Negative (N = 136)	80.9	**0.020**	90.4	0.163
	Positive (N = 91)	92.3		95.6	
PD-L2	Negative (N = 119)	81.5	0.086	90.8	0.296
	Positive (N = 108)	89.8		94.4	
IDO	Negative (N = 73)	84.9	0.823	93.2	0.882
	Positive (N = 154)	85.7		92.2	
TIM3	Negative (N = 123)	82.1	0.100	90.2	0.149
	Positive (N = 104)	89.4		95.2	
OX40	Negative (N = 143)	84.6	0.564	91.6	0.452
	Positive (N = 84)	86.9		94.0	
OX40L	Negative (N = 124)	80.6	**0.020**	90.3	0.157
	Positive (N = 103)	91.3		95.1	
B7-H2	Negative (N = 77)	83.1	0.502	90.9	0.523
	Positive (N = 150)	86.7		93.3	

*P* values in bold indicate statistical significance (*p* < 0.05). PFS, progression-free survival; OS, overall survival

### Prognostic significance of combined immune marker expression (combined immune score, CIS) in TCs and ICs

**Fig. 3:**
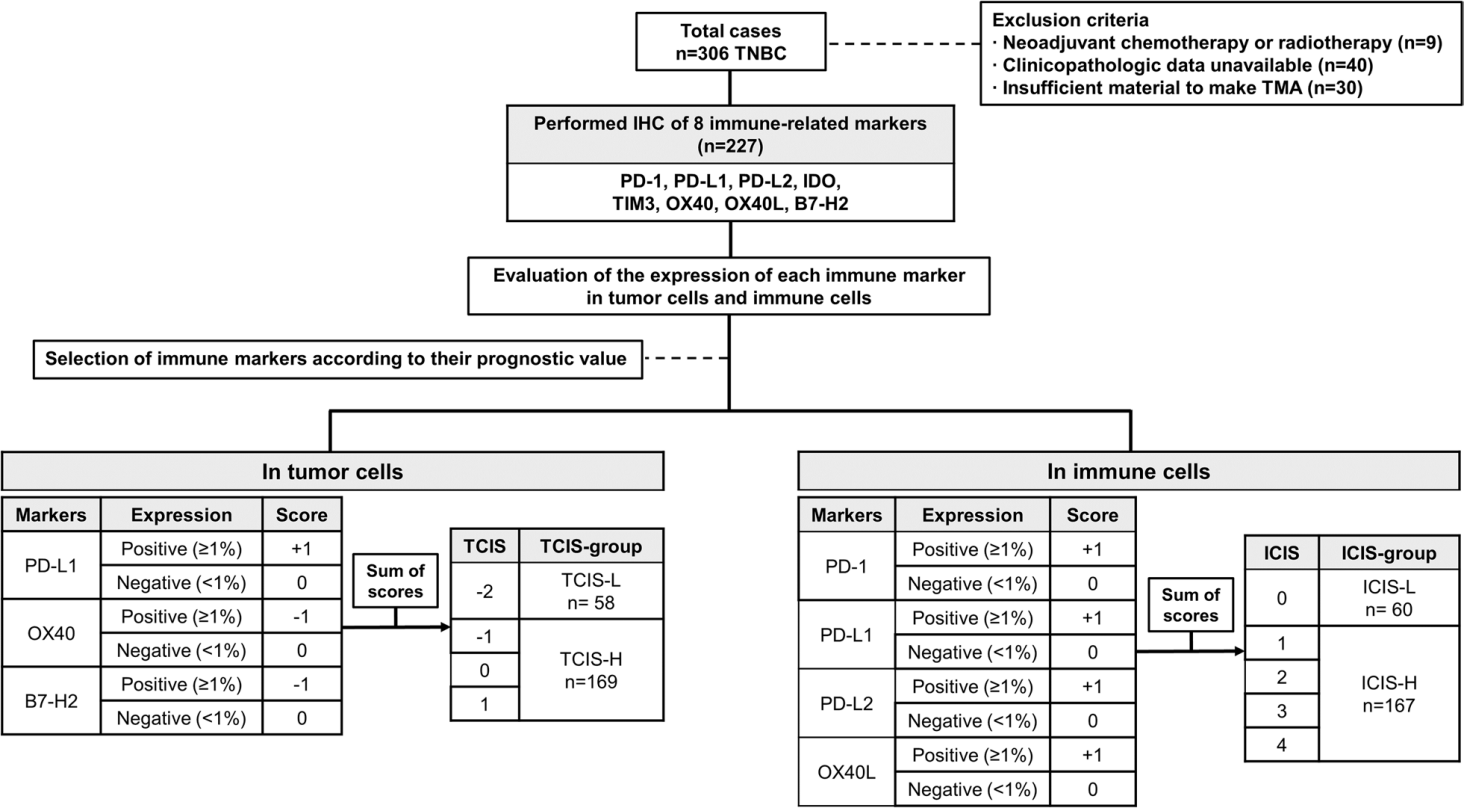
Flowchart showing the enrollment of TNBC patients and definition of combined immune scores (CIS) used in this study. TNBC, triple-negative breast cancer; TMA, tissue microarray; IHC, immunohistochemistry; TCIS, tumor cells combined immune score; TCIS-L, tumor cells combined immune score-low; TCIS-H, tumor cells combined immune score-high; ICIS, immune cells combined immune score; ICIS-L, immune cells combined immune score-low, ICIS-H, immune cells combined immune score-high

To comprehensively analyze the prognostic significance of combined immune marker expression in TNBC, we evaluated “combined immune scores (CIS)” expressed in both TCs and ICs. The definition of CIS and its scoring method in our study are summarized in Fig. 3. As the positive expression of all immune markers in both TCs and ICs failed to exhibit prognostic value (*p* < 0.1) regarding PFS and OS, CIS were constructed separately for TCs and ICs. First, we selected the three immune markers (PD-L1, OX40, and B7-H2) that showed prognostic significance (*p* < 0.1) in TCs, according to our previous results (Table 3). The expression of the three markers was counted as follows: one positive number (+ 1) for PD-L1 overexpression, as it was linked with a favorable prognosis, and one negative number (− 1) for OX40 and B7-H2 overexpression, both linked with unfavorable clinical outcomes. Then, the sum of the three immune markers was combined, and each TNBC patient was classified into a “tumor cells combined immune score—high (TCIS-H)” or a “tumor cells combined immune score—low (TCIS-L)” group, based on the cut-off of the relevant combined score (TCIS-H: ≥ − 1 and TCIS-L: <  − 1). In ICs, four immune markers (PD1, PD-L1, PD-L2, and OX40L) were included, based on our previous results (Table 3). The positive expression of all four immune markers was associated with a favorable PFS, thus each was counted as + 1. The sum of four immune markers was then calculated and each TNBC patient was classified within an “immune cells combined immune score—high (ICIS-H)” or an “immune cells combined immune score—low (ICIS-L)” group, based on the cut-off of the relevant combined score (ICIS-H: ≥ 1 and ICIS-L: < 1).

Of the 227 TNBC patients, 169 (74.4%) and 167 (73.6%) were classified as TCIS-H and ICIS-H, respectively (Additional file 5: Fig. S3). The clinicopathologic parameters according to the CIS groups are shown in Table 4. The TCIS-H group was significantly associated with a patient age 60 or older (*p* = 0.029), nuclear score 3 (*p* = 0.001), histologic grade 3 (*p* = 0.002), absence of ductal carcinoma in situ (*p* < 0.001), absence of lymphovascular invasion and lymph node metastasis (*p* = 0.003 and *p* = 0.017, respectively), and anatomic stage I & II (*p* < 0.001). The ICIS-H group was associated with a tumor size 2 cm or less (*p* = 0.037), histologic grade 3 (*p* = 0.011), absence of ductal carcinoma in situ (*p* = 0.011), absence of lymphovascular invasion (*p* = 0.019), and prognostic stage I & II (*p* = 0.031).

**Table 4:** Clinicopathologic parameters according to the combined immune score (CIS) groups

Variables	Combined immune score (CIS)					
	TCIS			ICIS		
	Low (n = 58)	High (n = 169)	*P* value	Low (n = 60)	High (n = 167)	*P* value
*Age (years)*						
< 60	55 (94.8%)	141 (83.4%)	**0.029**	48 (80.0%)	148 (88.6%)	0.095
≥ 60	3 (5.2%)	28 (16.6%)		12 (20.0%)	19 (11.4%)	
*Tumor size (cm)*						
≤ 2	20 (34.5%)	62 (36.7%)	0.763	15 (25.0%)	67 (40.1%)	**0.037**
> 2	38 (65.5%)	107 (63.3%)		45 (75.0%)	100 (59.9%)	
*Nuclear score*						
Score 1 & 2	20 (34.5%)	25 (14.8%)	**0.001**	17 (28.3%)	28 (16.8%)	0.054
Score 3	38 (65.5%)	144 (85.2%)		43 (71.7%)	139 (83.2%)	
*Histologic grade*						
Grade 1 & 2	18 (31.0%)	22 (13.0%)	**0.002**	17 (28.3%)	23 (13.8%)	**0.011**
Grade 3	40 (69.0%)	147 (87.0%)		43 (71.7%)	144 (86.2%)	
*Ductal carcinoma in situ*						
Absent	13 (22.4%)	83 (49.1%)	** < 0.001**	17 (28.3%)	79 (47.3%)	**0.011**
Present	45 (77.6%)	86 (50.9%)		43 (71.7%)	88 (52.7%)	
*Lymphovascular invasion*						
Absent	25 (43.1%)	110 (65.1%)	**0.003**	28 (46.7%)	107 (64.1%)	**0.019**
Present	33 (56.9%)	59 (34.9%)		32 (53.3%)	60 (35.9%)	
*Lymph node metastasis*						
Absent	32 (55.2%)	122 (72.2%)	**0.017**	40 (66.7%)	114 (68.3%)	0.820
Present	26 (44.8%)	47 (28.8%)		20 (33.3%)	53 (31.7%)	
*Anatomic stage*						
Stage I & II	37 (63.8%)	152 (89.9%)	** < 0.001**	48 (80.0%)	141 (84.4%)	0.430
Stage III	21 (36.2%)	17 (10.1%)		12 (20.0%)	26 (15.6%)	
*Prognostic stage*						
Stage I & II	15 (25.9%)	47 (27.8%)	0.774	10 (16.7%)	52 (31.1%)	**0.031**
Stage III	43 (74.1%)	122 (72.2%)		60 (83.3%)	115 (68.9%)	

*P* values in bold indicate statistical significance (*p* < 0.05). TCIS, tumor cells combined immune score; ICIS, immune cells combined immune score

**Fig. 4:**
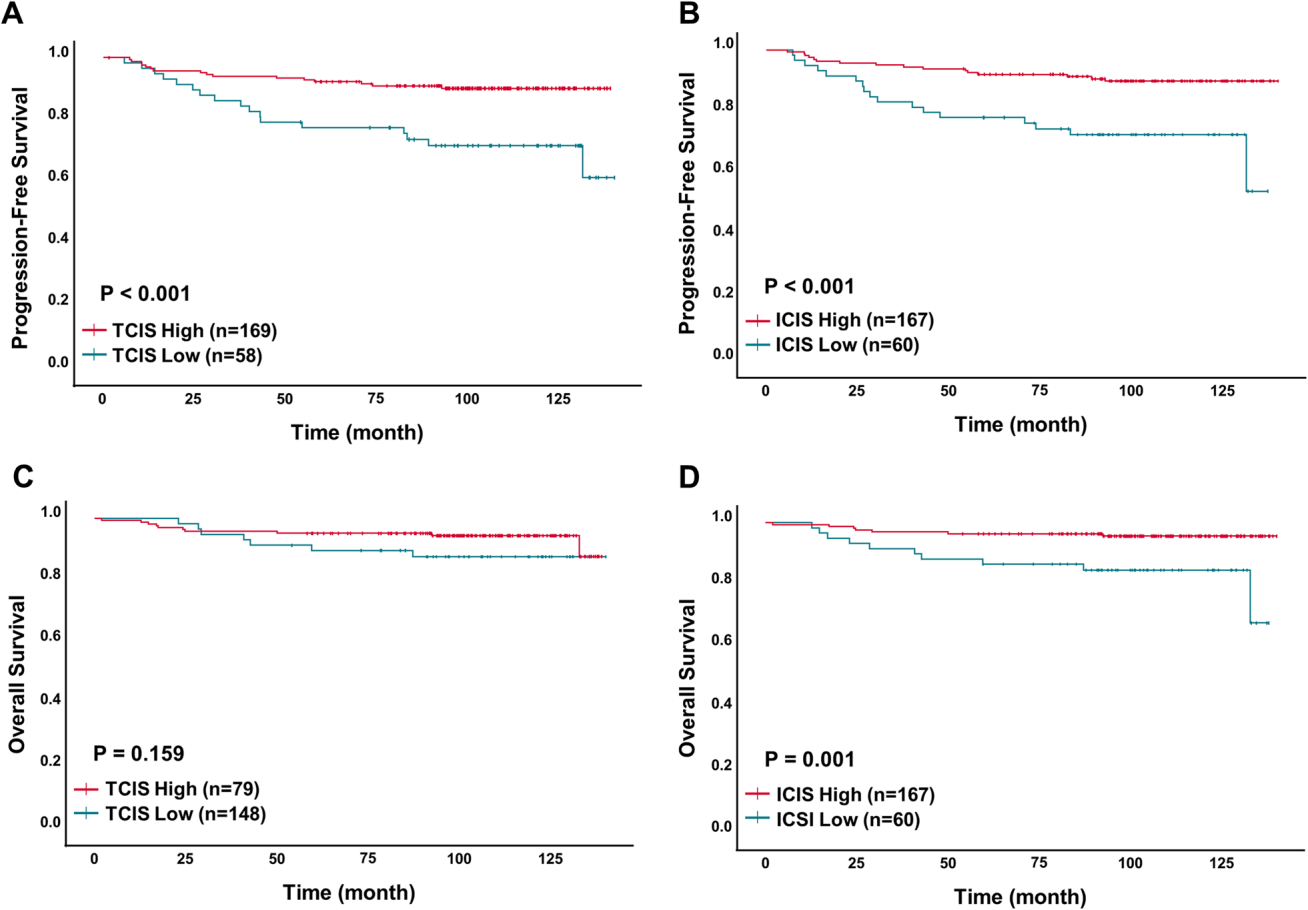
Kaplan–Meier analysis of progression-free survival (PFS) and overall survival (OS), according to the tumor cells combined immune score (TCIS) and immune cells combined immune score (ICIS) groups. **A** PFS in TCIS, **B**PFS in ICIS, **C** OS in TCIS, and **D** OS in ICIS

Of interest, both TCIS-H and ICIS-H were significantly associated with a longer PFS [(TCIS-H, 90.5% (mean 128.8 months) vs. TCIS-L, 70.7% (mean 110.8 months); *p* < 0.001) and ICIS-H group (ICIS-H, 90.4% (mean 130.0 months) vs. ICIS-L, 71.7% (mean 108.4 months); *p* < 0.001)] (Fig. 4A, B). On the other hand, only the ICIS-H group was associated with a longer OS compared to the ICIS-L group [ICIS-H, 95.8% (mean 135.4 months) vs. ICIS-L, 83.3% (mean 121.5 months); *p* = 0.001] (Fig. 4C, D).

**Fig. 5:**
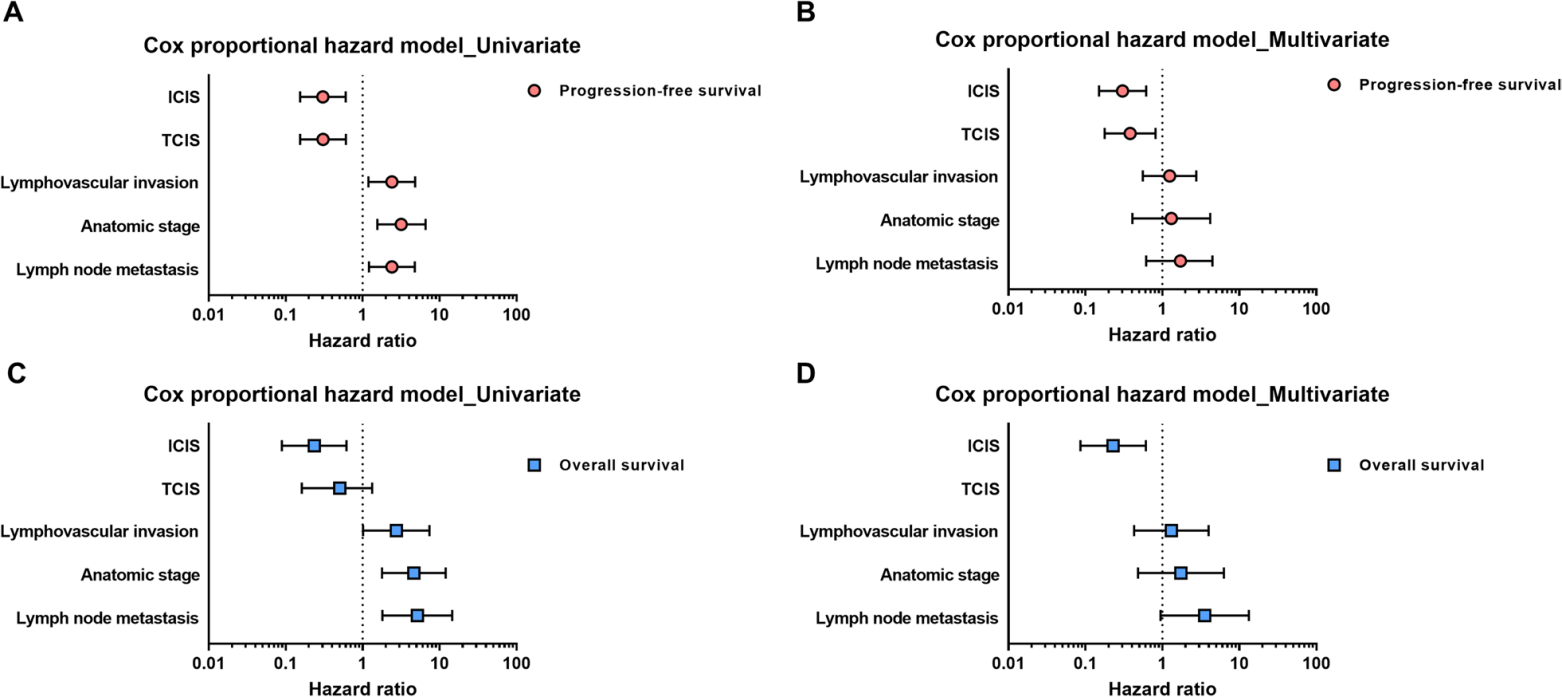
Univariate and multivariable Cox proportional hazard models to analyze the prognostic significance of the combined immune score (CIS) groups along with other clinicopathological variables. **A** and **B** progression-free survival (PFS), **C** and **D** overall-survival (OS)

As both TCIS and ICIS exhibited prognostic significance, we performed univariate and multivariable analysis along with other clinicopathological variables, using a Cox proportional hazard model, to determine whether TCIS and/or ICIS were independent prognostic predictors (Table 5; Fig. 5). Multivariate analysis identified both TCIS and ICIS as independent prognostic factors linked with PFS. More specifically, both TCIS-H and ICIS-H groups showed a significantly lower risk of progression compared with TCIS-L and ICIS-L groups [hazard ratio (HR) for TCIS = 0.381; 95% confidence interval (CI), 0.178–0.812; *p* = 0.012; HR for ICIS = 0.303; 95% CI 0.150–0.615; *p* = 0.001]. ICIS-H was also shown to be an independent factor predicting a lower risk of death compared with ICIS-L (HR = 0.229; 95% CI 0.086–0.609; *p* = 0.003).

**Table 5:** Univariate and multivariate analysis of progression-free survivals and overall survivals

	Progression-free survival				Overall survival			
	Univariate		Multivariate		Univariate		Multivariate	
Variables	HR (95% CI)	*P* value	HR (95% CI)	*P* value	HR (95% CI)	*P* value	HR (95% CI)	*P* value
*Lymphovascular invasion*								
Present	2.392 (1.189–4.809)	**0.014**	1.242 (0.558- 2.763)	0.596	2.738 (1.013–7.405)	**0.047**	1.308 (0.429–3.990)	0.637
Absent	1		1		1		1	
*Lymph node metastasis*								
Present	2.399 (1.207–4.747)	**0.012**	1.715 (0.616- 4.476)	0.302	5.137 (1.804–14.613)	**0.002**	3.545 (0.948–13.251)	0.060
Absent	1		1		1		1	
*Anatomic stage*								
III	3.163 (1.551–6.560)	**0.002**	1.305 (0.407- 4.188)	0.654	4.631 (1.784–12.022)	**0.002**	1.740 (0.482–6.289)	0.398
I&II	1		1		1		1	
*TCIS*								
High	0.305 (0.154–0.605)	**0.001**	0.381 (0.178–0.812)	**0.012**	0.505 (0.162–1.330)	0.167		
Low	1		1		1			
*ICIS*								
High	0.304 (0.154–0.602)	**0.001**	0.303 (0.150–0.615)	**0.001**	0.235 (0.089–0.616)	**0.003**	0.229 (0.086–0.609)	**0.003**
Low	1		1		1			

*P* values in bold indicate statistical significance (*p* < 0.05). HR, hazard ratio; CI, confidence interval; TCIS, tumor cells combined immune score; ICIS, immune cells combined immune score

### Validation of CIS

**Fig. 6:**
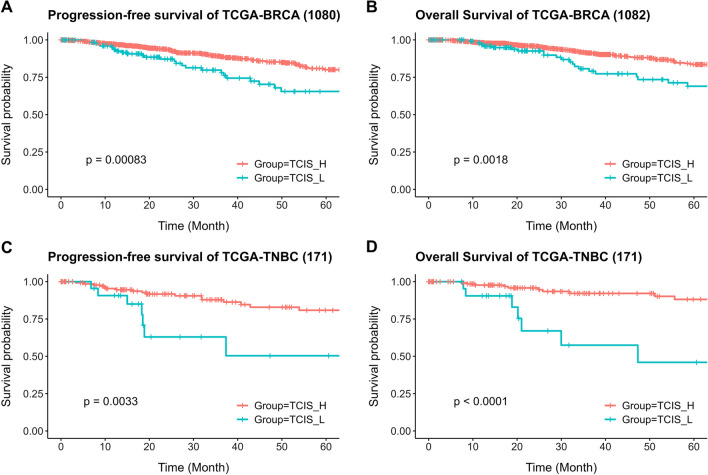
The progression-free survival (PFS) and overall survival (OS) of tumor cells combined immune score (TCIS) groups, according to the combination of mRNA expression levels of PD-L1, B7-H2, and OX40 retrieved from TCGA database. **A** PFS in breast cancer, **B** OS in breast cancer, **C** PFS in TNBC, and **D** OS in TNBC. TCGA, The Cancer Genome Atlas; BRCA, breast cancer; TNBC, triple-negative breast cancer

To further validate our findings regarding the prognostic role of TCIS, we extracted and tested the mRNA expression levels of the relevant immune markers from the TCGA database. In total, data from 1,082 breast cancers, including 171 TNBC samples, were extracted. The samples were classified into TCIS-H and TCIS-L groups according to the sum of PD-L1, B7-H2, and OX40 gene expression scores, following our immunohistochemical analysis. In total, 942 breast cancers were grouped as TCIS-H and 140 as TCIS-L, while the TCIS-H group was significantly associated with a longer 5-year PFS (*p* < 0.001) and 5-year OS (*p* = 0.0018) (Fig. 6a, b). In the TNBC cohort, the TCIS-H group also showed a significant association with a longer 5-year PFS (*p* = 0.0033) and 5-year OS (*p* < 0.001) (Fig. 6c, d), being concordant with the findings derived from our immunohistochemical analysis. In contrast to TNBC, the TCIS-H group was not significantly associated with PFS and OS in the other breast cancer subtypes (Additional file 6: Fig. S4).

## Discussion

In this study, we propose two combined immune score systems, the TCIS and ICIS, as novel prognostic models in TNBC. Eight immune markers of already established or potential prognostic or predictive value, including PD-L1, were evaluated [4, 38, 39]. PD-L1 was selected as a significant variable within both TCIS and ICIS models of the present study. The prognostic significance of PD-L1 in breast cancer is still unclear [40]. In general, breast cancers expressing PD-L1 have been linked with shorter survival, while PD-L1 overexpression in TILs may be an indicator of favorable prognosis [41, 42]. However, other studies have reported that PD-L1 overexpression on tumor or stromal cells may be associated with a longer survival in TNBC [43–45]. Additionally, PD-L1 positive expression has been reported as a biomarker predicting response to ICI treatment in advanced TNBC patients, although this expression may be a predictor of response to chemotherapy and not associated with ICI benefit in early TNBCs [46–49]. In our study, PD-L1 positive expression in ICs was linked with a longer PFS; likewise, PD-L1 overexpression in TCs also showed a favorable prognostic association trend. Interestingly, in addition to single immune marker testing, TCIS-H was also associated with a favorable prognosis, a result validated through the examination of relevant mRNA expression levels from TCGA database. Besides supporting the current evidence regarding PD-L1 immunohistochemical interpretation in TCs and ICs, our study additionally highlights the need for further research to elucidate the prognostic role of other immune markers, especially in TNBC.

Even though the expression of PD-L1 is a crucial predictive factor for the treatment with PD-1/L1 blockade, many recent studies emphasize the importance of deciphering the complex interaction among several types of immune cells and tumor cells to overcome the low rate of therapeutic response. In the present study, we enrolled eight immune checkpoint-related markers and suggested two CIS predicting prognosis in TNBC. Both TCIS and ICIS were identified as independent prognostic factors of PFS in our multivariate analysis. More specifically, the TCIS-H and ICIS-H groups were linked with a longer PFS, while the ICIS-H group was additionally associated with a longer OS. These results reflect that the CIS models may depict more comprehensively the complex immunologic characteristics of TNBC and predict a patient’s prognosis more robustly than the expression of a single checkpoint. Besides PD-1/PD-L1, several other immune checkpoints have recently been under investigation for their potential therapeutic value in TNBC, and any combination of regimens, possibly together with the PD-1/PD-L1 blockade approach, might be more efficacious than a single ICI [9]. Therefore, the value of a multi-biomarker panel, rather than testing a single biomarker, such as PD-L1, should be considered and further investigated. In addition, our novel findings regarding TCIS and ICIS may support the evidence of performing multiple protein-based tests, before administering a combined immunotherapy in TNBC patients.

In this study, we defined the positive group when the staining was present in greater than or equal to 1% of both TCs and ICs, based on already published PD-L1 scoring formulas (36, 50, 51). The combined positive score (CPS) using the 22C3 clone is currently the only approved PD-L1 scoring algorithm in TNBC [5]. CPS is complex and often not reproducible [52], whereas the 1% cutoff in scoring tumor or immune cells of various cancers [2] is simpler to implement in routine pathology practice. Apart from predicting response to ICIs in various malignancies, the 1% cut-off is also widely used by pathologists to evaluate estrogen/progesterone expression and identify suitable candidates for hormone therapy in breast cancer [32, 53]. Currently, most companion diagnostics for targeted cancer therapies are based on immunohistochemistry rather that high-throughput genetic analysis, due to cost-effectiveness and easy accessibility with rapid turnaround times [19, 21]. Along with PD-L1, the most representative immune checkpoint molecule, the remaining seven markers were also evaluated with immunohistochemistry in our study, aiming to highlight the potential value of a CIS in the clinical setting. We anticipate that our immunohistochemical scoring system might be applied as a novel immune-based prognostic model in TNBC.

## Conclusion

By analyzing eight immune markers in TNBC with immunohistochemistry, we highlighted the significance of testing multiple biomarkers and providing combined immune scores. Both TCIS and ICIS exhibited a significant prognostic value in TNBC patients in our study. However, further studies would be needed to identify and implement the most efficient prognostic and predictive biomarkers of immunotherapy in the management of TNBC patients.

### Supplementary Information

**Additional file 1. Table S1.** Expression of the tested immune-related markers in both tumor and immune cells, and its clinicopathological significance.

**Additional file 2. Table S2.** Prognostic role of the expression of each immune marker in both tumor and immune cells.

**Additional file 3. Fig. S1.** Expression of each immune marker in tumor cells and its prognostic significance. Kaplan Meier graph for progression-free survival (PFS) and overall survival (OS). (A and B) in PD-L1; (C and D) in PD-L2; (E and F) in IDO; (G and H) in TIM3; (I and J) in OX40; (K and L) in OX40L and (M and N) in B7-H2.

**Additional file 4. Fig. S2.** Expression of each immune marker in immune cells and its prognostic significance. Kaplan Meier graph for progression-free survival (PFS) and overall survival (OS). (A and B) in PD-1; (C and D) in PD-L1; (E and F) in PD-L2; (G and H) in IDO; (I and J) in TIM3; (K and L) in OX40; (M and N) in OX40L and (O and P) in B7-H2.

**Additional file 5. Fig. S3.** Grouped by combined immune scores (CIS). In tumor cells combined immune score (TCIS), 169 cases were classified as tumor cells combined immune score-high (TCIS-H) and 58 cases as tumor cells combined immune score-low (TCIS-L). In immune cells combined immune score (ICIS), 167 cases were classified as immune cells combined immune score-high (ICIS-H) and 60 cases as immune cells combined immune score-low (ICIS-L).

**Additional file 6. Fig. S4.** The progression-free survival (PFS) and overall survival (OS) of tumor cells combined immune score (TCIS) groups, according to the combination of mRNA expression levels of PD-L1, B7-H2, and OX40 in hormone receptor-positive (HRP) and HER2-positive (HER2) breast cancer from TCGA database. (A) PFS in HRP breast cancer, (B) OS in HRP breast cancer, (C) PFS in HER2-positive breast cancer, and (D) OS in HER2-positive breast cancer. Abbreviations: TCGA, The Cancer Genome Atlas; HRP, hormone receptor positive breast cancer; HER2, HER2 positive breast cancer.

## Data Availability

The data generated or analyzed during this study can be made available by the corresponding author upon reasonable request.

## Abbreviations

ICIs: Immune checkpoint inhibitors
CTLA-4: Cytotoxic T lymphocyte antigen-4
PD-1: Programmed death 1
PD-L1: Programmed death ligand 1
TNBC: Triple-negative breast cancer
FDA: Food and Drug Administration
TCGA: The Cancer Genome Atlas
TCs: Tumor cells
ICs: Immune cells
CIS: Combined immune scores
AJCC: American Joint Committee on Cancer
ER: Estrogen receptor
PR: Progesterone receptor
HER2: Human epidermal growth factor receptor 2
FISH: Fluorescence in situ hybridization
IS: Intensity score
PS: Proportion score
PFS: Progression-free survival
OS: Overall survival
TCIS-H: Tumor cells combined immune score—high
TCIS-L: Tumor cells combined immune score—low
ICIS-H: Immune cells combined immune score—high
ICIS-L: Immune cells combined immune score—low
HR: Hazard ratio
CI: Confidence interval
CPS: Combined positive score
