# Supplementary material for: The prognostic value of a combined immune score in tumor and immune cells assessed by immunohistochemistry in triple-negative breast cancer
Source: Breast Cancer Res. 2023 Nov 3;25:134. doi: 10.1186/s13058-023-01710-8 (PMC10625207; doi:10.1186/s13058-023-01710-8)
Supplement: Supplementary file 1 — Additional file 1. Table S1. Expression of the tested immune-related markers in both tumor and immune cells, and its clinicopathological significance. [file 13058_2023_1710_MOESM1_ESM.docx]

Table S1. Expression of the tested immune-related markers in both tumor and immune cells, and its clinicopathological significance.

| Expression of immune markers | Lymph node metastasis | | | | Anatomic stage | | | Prognostic stage | | | |
| --- | --- | --- | --- | --- | --- | --- | --- | --- | --- | --- | --- |
|  | Absent  (N=154) | Present  (N=73) | *P* value | | Ⅰ&Ⅱ  (N=189) | Ⅲ  (N=38) | *P* value | Ⅰ&Ⅱ  (N=62) | Ⅲ  (N=165) | *P* value | |
| In both tumor and immune cells |  |  | |  |  |  |  |  |  | |  |
| PD-1 |  |  | |  |  |  |  |  |  | |  |
| Positive (N=0) | 0 (0.0%) | 0 (0.0%) | |  | 0 (0.0%) | 0 (0.0%) |  | 0 (0.0%) | 0 (0.0%) | |  |
| Negative (N=227) | 154 (67.8%) | 73 (32.2%) | | - | 189 (83.3%) | 38 (16.7%) | - | 62 (27.3%) | 165 (72.7%) | | - |
| PD-L1 |  |  | | 0.544 |  |  | - |  |  | | 0.606 |
| Positive (N=46) | 19 (73.1%) | 7 (26.9%) | |  | 24 (92.3%) | 2 (7.7%) |  | 6 (23.1%) | 20 (76.9%) | |  |
| Negative (N=181) | 135 (67,2%) | 66 (32.8%) | |  | 165 (82.1%) | 36 (17.9%) |  | 56 (27.9%) | 145 (72.1%) | |  |
| PD-L2 |  |  | | 0.810 |  |  | 0.180 |  |  | | 0.420 |
| Positive (N=197) | 67 (67.0%) | 33 (33.0%) | |  | 87 (87.0%) | 13 (13.0%) |  | 30 (30.0%) | 70 (70.0%) | |  |
| Negative (N=30) | 87 (68.5%) | 40 (31.5%) | |  | 102 (30.3%) | 25 (19.7%) |  | 32 (25.2%) | 95 (74.8%) | |  |
| IDO |  |  | | 0.142 |  |  | 0.482 |  |  | | 0.298 |
| Positive (N=129) | 56 (62.2%) | 34 (37.8%) | |  | 73 (81.1%) | 17 (18.9%) |  | 28 (31.1%) | 62 (68.9%) | |  |
| Negative (N=98)_ | 98 (71.5%) | 39 (28.5%) | |  | 116 (84.7%) | 21 (15.3%) |  | 34 (24.8%) | 103 (75.2%) | |  |
| TIM3 |  |  | | 0.053 |  |  | 0.296 |  |  | | 0.327 |
| Positive (N=109) | 36 (58.1%) | 26 (41.9%) | |  | 49 (79.0%) | 13 (21.0%) |  | 14 (22.6%) | 48 (77.4%) | |  |
| Negative (N=118) | 118 (71.5%) | 47 (28.5%) | |  | 140 (84.8%) | 25 (15.2%) |  | 48 (29.1%) | 117 (70.9%) | |  |
| OX40 |  |  | | **0.005** |  |  | **<0.001** |  |  | | 0.701 |
| Positive (N=82) | 22 (50.0%) | 22 (50.0%) | |  | 27 (61.4%) | 17 (38.6%) |  | 11 (25.0%) | 33 (75.0%) | |  |
| Negative (N=145) | 132 (72.1%) | 51 (27.9%) | |  | 162 (88.5%) | 21 (11.5%) |  | 51 (27.9%) | 132 (72.1%) | |  |
| OX40L |  |  | | 0.263 |  |  | 0.227 |  |  | | 0.343 |
| Positive (N=86) | 30 (61.2%) | 19 (38.8%) | |  | 38 (77.6%) | 11 (22.4%) |  | 16 (32.7%) | 33 (67.3%) | |  |
| Negative (N=141) | 124 (69.7%) | 54 (30.3%) | |  | 151 (84.8%) | 27 (15.2%) |  | 46 (25.8%) | 132 (74.2%) | |  |
| B7-H2 |  |  | | 0.306 |  |  | 0.745 |  |  | | 0.535 |
| Positive (N=193) | 86 (65..2%) | 46 (34.8%) | |  | 109 (82.6%) | 23 (17.4%) |  | 34 (25.8%) | 98 (74.2%) | |  |
| Negative (N=34) | 68 (71.6%) | 27 (28.4%) | |  | 80 (84.2%) | 15 (15.8%) |  | 28 (29.5%) | 67 (70.5%) | |  |

*P* values in bold indicate statistical significance (p < 0.05).
