# Supplementary material for: The prognostic value of a combined immune score in tumor and immune cells assessed by immunohistochemistry in triple-negative breast cancer
Source: Breast Cancer Res. 2023 Nov 3;25:134. doi: 10.1186/s13058-023-01710-8 (PMC10625207; doi:10.1186/s13058-023-01710-8)
Supplement: Supplementary file 2 — Additional file 2. Table S2. Prognostic role of the expression of each immune marker in both tumor and immune cells. [file 13058_2023_1710_MOESM2_ESM.docx]

Table S2. Prognostic role of the expression of each immune marker in both tumor and immune cells

| Immune markers expression |  | PFS (%) | *P* value | OS (%) | *P* value |
| --- | --- | --- | --- | --- | --- |
| PD-1 | Positive (N=0) | - | - |  | - |
|  | Negative (N=227) | 85.5% |  | 92.5% |  |
| PD-L1 | Positive (N=26) | 92.3% | 0.297 | 96.2% | 0.475 |
|  | Negative (N=201) | 84.6% |  | 92.0% |  |
| PD-L2 | Positive (N=100) | 89.0% | 0.198 | 94.0% | 0.456 |
|  | Negative (N=127) | 82.7% |  | 91.3% |  |
| IDO | Positive (N=90) | 85.6% | 0.864 | 94.4% | 0.351 |
|  | Negative (N=137) | 85.4% |  | 91.2% |  |
| TIM3 | Positive (N=62) | 87.1% | 0.637 | 93.5% | 0.704 |
|  | Negative (N=165) | 84.8% |  | 92.1% |  |
| OX40 | Positive (N=44) | 84.1% | 0.857 | 95.5% | 0.383 |
|  | Negative (N=183) | 85.8% |  | 91.8% |  |
| OX40L | Positive (N=49) | 89.8% | 0.313 | 95.9% | 0.313 |
|  | Negative (N=178) | 84.3% |  | 91.6% |  |
| B7-H2 | Positive (N=132) | 84.8% | 0.754 | 92.4% | 0.983 |
|  | Negative (N=95) | 86.3% |  | 92.6% |  |

PFS, progression-free survival; OS, overall survival
