# Supplementary figures and images for: The prognostic value of a combined immune score in tumor and immune cells assessed by immunohistochemistry in triple-negative breast cancer
Source: Breast Cancer Res. 2023 Nov 3;25:134. doi: 10.1186/s13058-023-01710-8 (PMC10625207; doi:10.1186/s13058-023-01710-8)

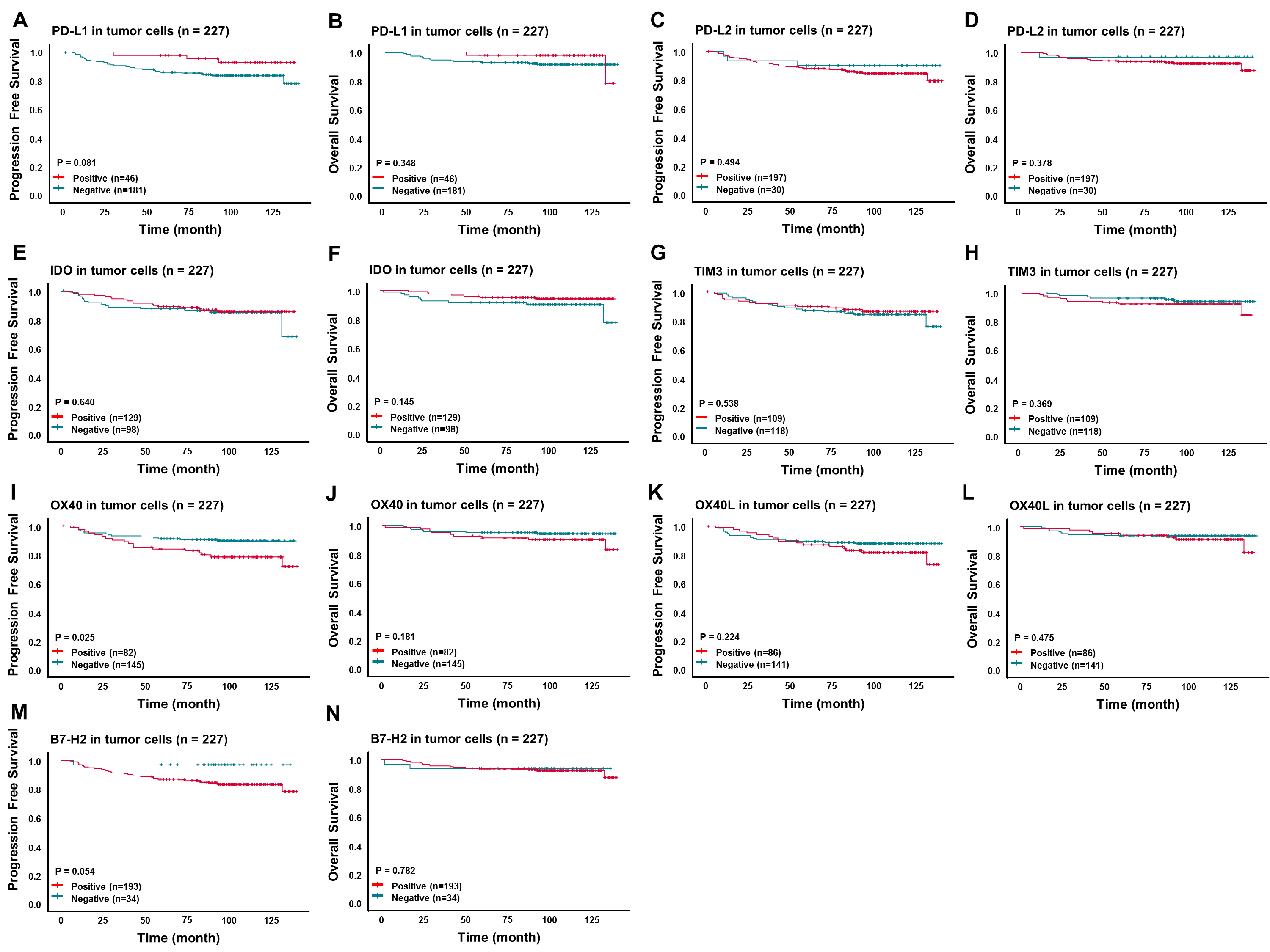

Supplement: Supplementary file 3 — Additional file 3. Fig. S1. Expression of each immune marker in tumor cells and its prognostic significance. Kaplan Meier graph for progression-free survival (PFS) and overall survival (OS). (A and B) in PD-L1; (C and D) in PD-L2; (E and F) in IDO; (G and H) in TIM3; (I and J) in OX40; (K and L) in OX40L and (M and N) in B7-H2. [file 13058_2023_1710_MOESM3_ESM.png]

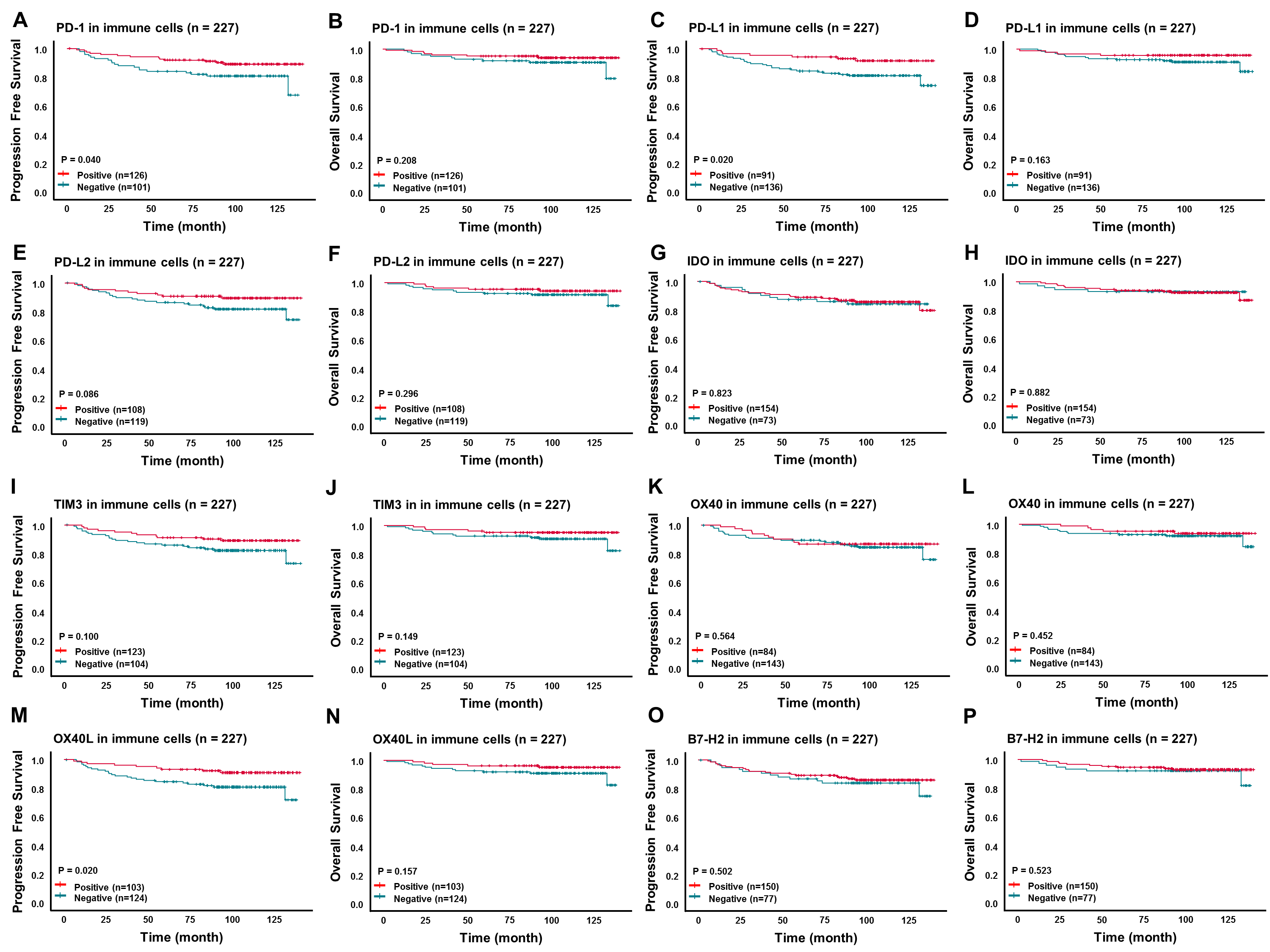

Supplement: Supplementary file 4 — Additional file 4. Fig. S2. Expression of each immune marker in immune cells and its prognostic significance. Kaplan Meier graph for progression-free survival (PFS) and overall survival (OS). (A and B) in PD-1; (C and D) in PD-L1; (E and F) in PD-L2; (G and H) in IDO; (I and J) in TIM3; (K and L) in OX40; (M and N) in OX40L and (O and P) in B7-H2. [file 13058_2023_1710_MOESM4_ESM.png]

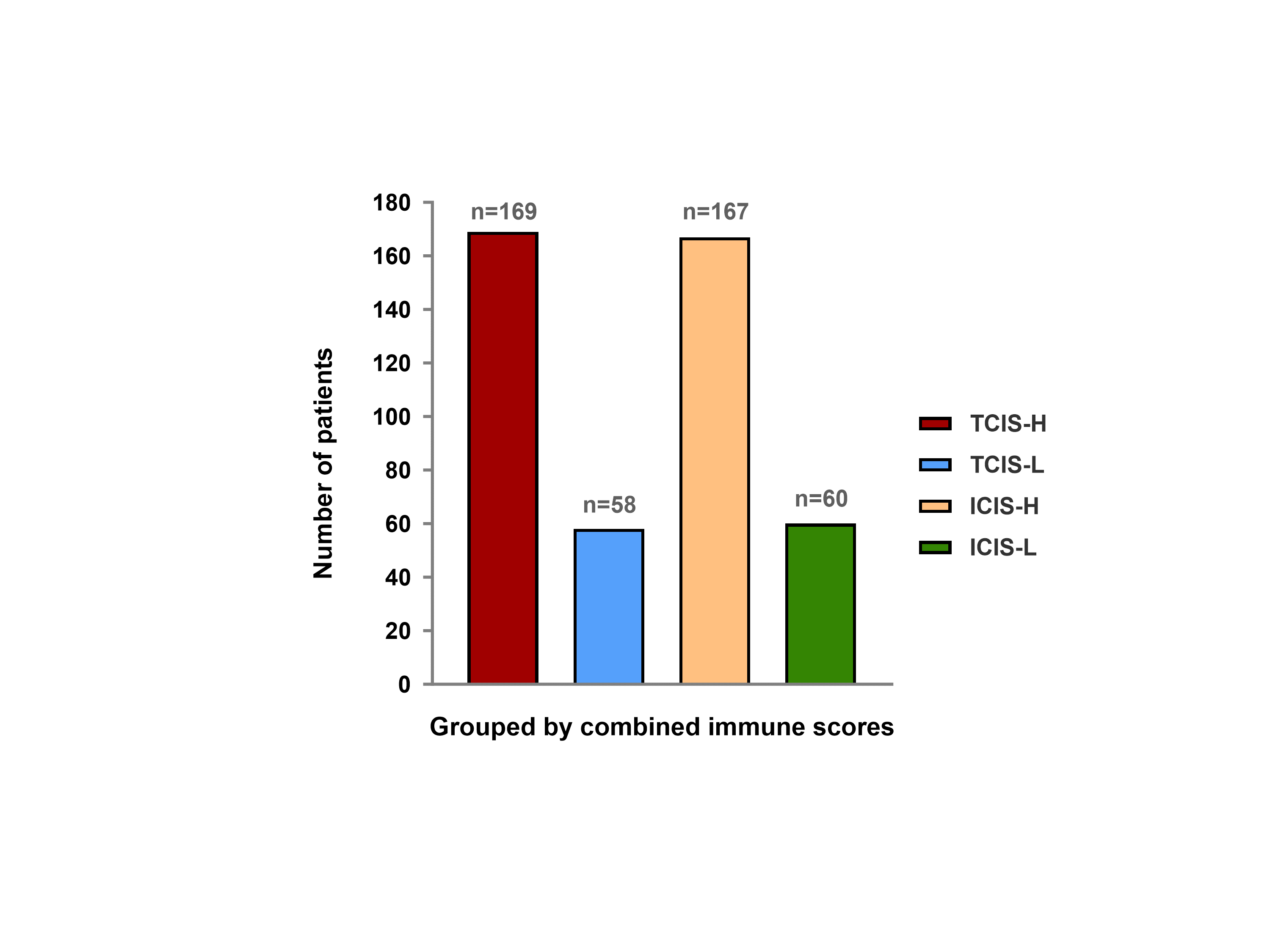

Supplement: Supplementary file 5 — Additional file 5. Fig. S3. Grouped by combined immune scores (CIS). In tumor cells combined immune score (TCIS), 169 cases were classified as tumor cells combined immune score-high (TCIS-H) and 58 cases as tumor cells combined immune score-low (TCIS-L). In immune cells combined immune score (ICIS), 167 cases were classified as immune cells combined immune score-high (ICIS-H) and 60 cases as immune cells combined immune score-low (ICIS-L). [file 13058_2023_1710_MOESM5_ESM.png]

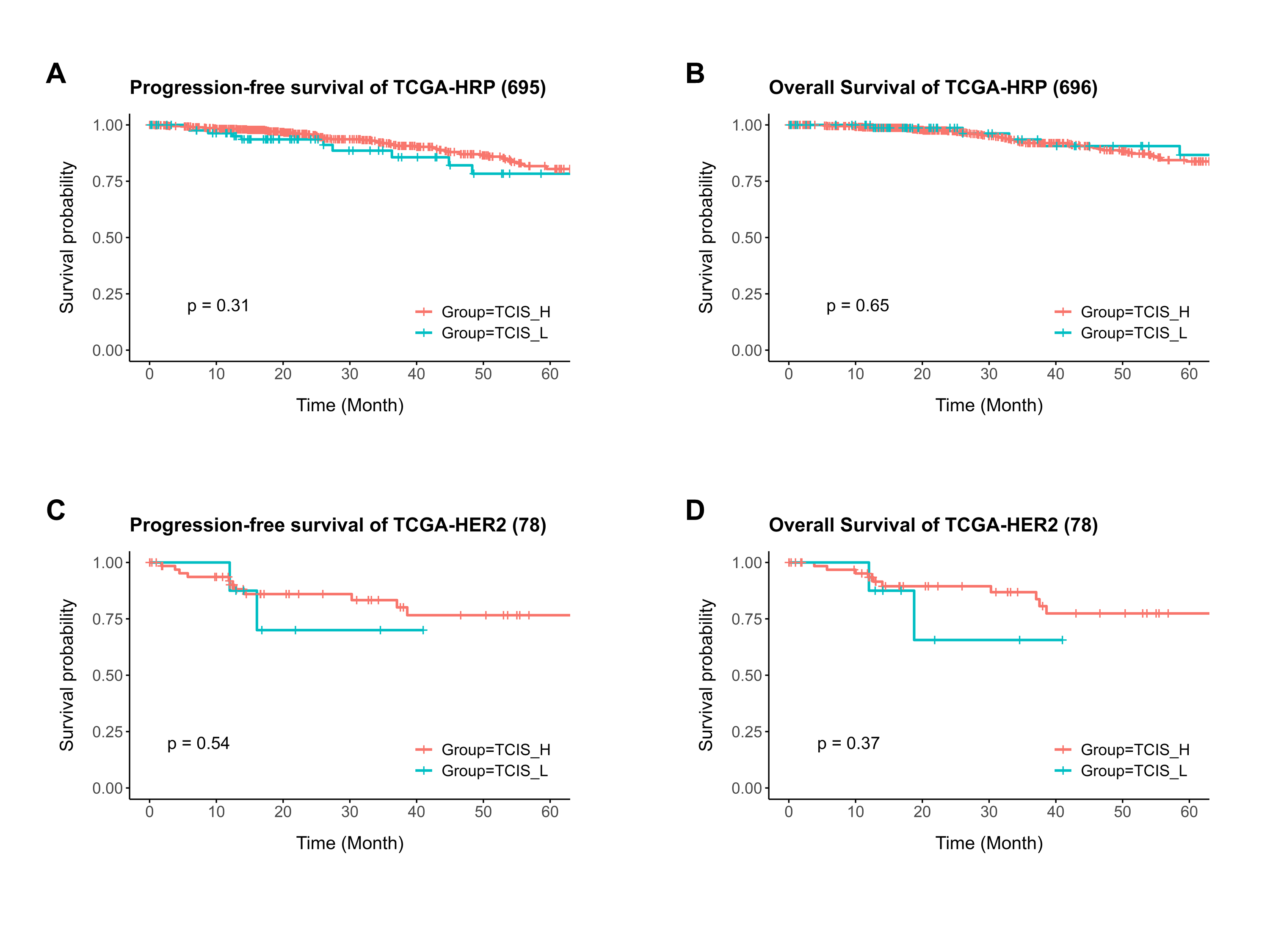

Supplement: Supplementary file 6 — Additional file 6. Fig. S4. The progression-free survival (PFS) and overall survival (OS) of tumor cells combined immune score (TCIS) groups, according to the combination of mRNA expression levels of PD-L1, B7-H2, and OX40 in hormone receptor-positive (HRP) and HER2-positive (HER2) breast cancer from TCGA database. (A) PFS in HRP breast cancer, (B) OS in HRP breast cancer, (C) PFS in HER2-positive breast cancer, and (D) OS in HER2-positive breast cancer. Abbreviations: TCGA, The Cancer Genome Atlas; HRP, hormone receptor positive breast cancer; HER2, HER2 positive breast cancer. [file 13058_2023_1710_MOESM6_ESM.png]
